# Lighting during grow-out and *Salmonella *in broiler flocks

**DOI:** 10.1186/1751-0147-52-46

**Published:** 2010-06-29

**Authors:** Victoriya V Volkova, J Allen Byrd, Sue Ann Hubbard, Danny Magee, Richard H Bailey, Robert W Wills

**Affiliations:** 1 Epidemiology group, Centre for Infectious Diseases, University of Edinburgh, R. 138, Ashworth Laboratories, Kings Buildings, West Mains Road, Edinburgh, EH9 3JT, UK; 2 USDA-ARS-SPARC, 2881 F&B Road, College Station, TX 77845, USA; 3 Department of Pathobiology and Population Medicine, College of Veterinary Medicine, Mississippi State University, P.O. Box 6100, Mississippi State, MS 39759, USA

## Abstract

**Background:**

Lighting is used during conventional broiler grow-out to modify bird behaviour to reach the goals of production and improve bird welfare. The protocols for lighting intensity vary. In a field study, we evaluated if the lighting practices impact the burden of *Salmonella *in broiler flocks.

**Methods:**

Conventional grow-out flocks reared in the states of Alabama, Mississippi and Texas, USA in 2003 to 2006 were sampled 1 week before harvest (*n *= 58) and upon arrival for processing (*n *= 56) by collecting feathered carcass rinsate, crop and one cecum from each of 30 birds, and during processing by collecting rinsate of 30 carcasses at pre-chilling (*n *= 56) and post-chilling points (*n *= 54). Litter samples and drag swabs of litter were collected from the grow-out houses after bird harvest (*n *= 56). Lighting practices for these flocks were obtained with a questionnaire completed by the growers. Associations between the lighting practices and the burden of *Salmonella *in the flocks were tested while accounting for variation between the grow-out farms, their production complexes and companies.

**Results:**

Longer relative duration of reduced lights during the grow-out period was associated with reduced detection of *Salmonella *on the exterior of birds 1 week before harvest and on the broiler carcasses at the post-chilling point of processing. In addition, starting reduced lights for ≥18 hours per day later in the grow-out period was associated with decreased detection of *Salmonella *on the exterior of broilers arriving for processing and in the post-harvest drag swabs of litter from the grow-out house.

**Conclusions:**

The results of this field study show that lighting practices implemented during broiler rearing can impact the burden of *Salmonella *in the flock. The underlying mechanisms are likely to be interactive.

## Background

A significant amount of research has been done to understand how lighting can be used to maximize performance of broiler breeders, and in grow-out broilers to achieve a balance between production and welfare goals. The grow-out lighting protocols for broiler flocks vary and are designed to maximize such production indexes as feed conversion, final weight of the bird carcass, and weights of individual carcass parts (breast, legs, wings) [1]. At the same time, lighting affects welfare of the birds, in particular activity rhythms and resting time, level of stress, pecking and scratching behaviour, and walking ability [2]. We hypothesize that by affecting the birds' physiology and behaviour the lighting practices may impact the distribution of food-borne pathogens such as *Salmonella *and *Campylobacter *in chicken flocks. In the present analysis we evaluate if/how the lighting practices during conventional broiler grow-out affect the burden of *Salmonella* in flocks during rearing, as well as on the carcasses in processing.

Broadly, the grow-out lighting can be classified as either 'constant lights' or 'intermittent lights'. With constant lights, full intensity lights are maintained for 24 hours per day during the entire grow-out period. With intermittent lights, the grow-out starts with several days of full lights for 24 hours per day, after which reduced (dim) or black-out (minimum intensity) lights are introduced. During the last 2/3 of the grow-out period, full lights may be applied for less than 6 hours per day, or not applied at all. During the last 2 to 3 days prior to bird harvest some broiler-growers maintain full lights for 24 hours per day, while others maintain dim lights at this time.

In a prospective field observational study, we measured the burden of *Salmonella *in conventional grow-out broiler flocks 1 week before the end of rearing, upon arrival for processing and during processing, and in the house litter after bird harvest. The grow-out lighting practices for sampled flocks were surveyed with a questionnaire completed by the growers. We then tested if the probabilities of detecting *Salmonella *in the birds, in the litter, and on the carcasses during processing were associated with parameters of the grow-out lighting.

## Materials and methods

### Selection and number of flocks

The flocks sampled were reared on 29 conventional grow-out broiler farms in the states of Alabama, Mississippi and Texas, USA during 2003 to 2006. The sample collection has been described previously [3]. In brief, two flocks were sampled per farm. Each sampled flock was reared in a single house. Sampled flocks were reared for ten broiler complexes of two companies. The farms to be sampled were selected by the companies prior to the flocks' placements so that the flocks would be processed at the start of a working week to facilitate laboratory processing of the samples. Despite the convenience sampling, we consider that the sampled flocks were generally representative of broilers reared in the area of the study.

Description of the grow-out lighting was obtained for each of 58 flocks sampled approximately 1 week before the end of rearing. At this time the birds were 41-57 days old, with an average of 49 days. Fifty-six of the flocks were sampled again upon arrival for processing and during processing prior to immersion into the chilling-water tank, and 54 of the flocks immediately after the chilling. At the time of harvest the birds were 48-61 days old, 56 days old on average, and a sampled flock numbered around 15,200-27,200 birds. Later in the day of bird harvest, the litter was sampled in 56 of the grow-out houses where the flocks were reared.

The Mississippi State University Institutional Animal Use and Care Committee gave approval of the project through IACUC Protocol #02-040 on July 15, 2002.

### Selection of birds and carcasses, and samples taken

Approximately 1 week before the end of rearing, a convenience sample of 30 broilers was selected from the flock from the cool-cell end of the house (the cool-cell is a built-in evaporative culling pad system). The birds were immediately euthanized by cervical dislocation, and the whole feathered carcass rinsate, crop, and ceca were obtained from each bird carcass. First, the rinsate was obtained by placing the carcass into a sterile bio-hazard bag with 250 mL of buffered peptone water (BPW), vigorously shaking the carcass in the bag for 1 minute, and aseptically transferring the rinsate into a sterile plastic bottle. After the rinsate was collected, the ceca and crop were aseptically removed from the carcass. Each cecum was placed into a sterile Whirl-Pak^® ^Bag and the crop into a sterile Whirl-Pak^® ^Filter Bag (NASCO, Fort Atkinson, WI, USA). Cecal and crop samples were processed in the field immediately after the sample collection. One cecum (either the left or the right) was retained for another research project. The other cecum was weighed and 9-times its weight of tetrathionate (TET) broth (Remel Inc., Lenexa, KS, USA) was added; the mixture was stomached for 60 seconds. To the crop sample 9-times its weight of BPW was added; this mixture was stomached for 60 seconds.

Upon flock's arrival at the processing plant, 5 cages were selected from each of the 3 livehaul trucks used to transport the flock from the farm. Two birds were removed from each cage, for a total of 30 broilers from the flock. The birds were immediately euthanized by cervical dislocation. The whole feathered carcass rinsate, cecal and crop samples from each bird carcass were collected and processed similarly to those obtained 1 week before the end of rearing.

During flock processing, the rinsates of 30 eviscerated carcasses (with feathers, head, and feet removed) were obtained immediately before the final carcass rinse prior to the immersion chilling. Immediately after the chilling tank, rinsates were obtained from 30 other carcasses from the flock. The collection of the carcass rinsates at each of the two points was timed so that it was evenly dispersed over the course of the flock passing through the point. That is, the first carcass was sampled at the beginning of the flock passing through, and then the other 29 carcasses were taken from the processing line at a repeating time interval adjusted for the speed of the line. Each carcass was aseptically removed from the processing line with newly gloved hands, placed into a sterile plastic bag with 100 mL of Butterfield's solution, and vigorously shaken in the bag for 1 minute; the rinsate was aseptically transferred into a sterile plastic bottle. To bring the final concentration to a single-strength BPW, 10 mL of 10-times concentrated BPW was added to the rinsate. After that 10 mL was removed from each bottle for another research project.

The processed samples from birds and carcasses were transported at room temperature and delivered to the laboratory within 8 hours of the sample collection.

### Sampling broiler litter

Later in the day after the flock harvest, 4 pooled litter samples and 4 drag swabs of litter were obtained from the grow-out house. The pooled litter samples were collected as described by Volkova *et al. *[4]. The drag swabs were prepared, collected, and processed as previously described [5-8]. The litter samples and drag swabs were collected over the full length and breadth of the grow-out house floor. These samples were transported to the laboratory on wet ice and delivered within 8 hours of the sample collection. Upon arrival, 25 grams of each pooled litter sample was placed into a Whirl-Pak^® ^Filter Bag; 225 mL of BPW was added and mixed for 1 minute. To each drag swab sample, 100 mL of BPW was added and mixed.

### *Salmonella *isolation and identification

Each cecal, crop or rinsate sample delivered to the laboratory was incubated at 42°C overnight. Each drag swab or litter sample processed in the laboratory immediately upon arrival was then incubated at 42°C overnight. *Salmonella *isolation from all the samples was performed similarly to that previously described [8,9]. In brief, after the overnight incubation, 1.0 mL of the sample was transferred to 9.0 mL of TET broth, vortexed and incubated at 42°C for 48 hours. After incubation, 0.1 mL of TET was transferred to 9.9 mL of Rappaport-Vassiliadis (RV) broth (DIFCO Laboratories, Detroit, MI, USA) and incubated at 42°C overnight. After incubation, one loopful of RV was plated onto a xylose-lysine-tergitol 4 (XLT4) agar plate (Remel Inc., Lenexa, KS, USA) and incubated at 37°C overnight. After incubation, the plates were examined for *Salmonella*-like colonies, and a single colony was picked from a *Salmonella*-positive XLT4 plate. *Salmonella *identity was confirmed by biochemical tests on Triple Sugar Iron and Lysine Iron Agar slants, and in a slide agglutination assay using *Salmonella *O Antiserum Poly A-I & Vi (DIFCO Laboratories, Detroit, MI, USA) as described by the manufacturer.

### Survey of lighting practices

The survey questionnaire was completed by owners or managers of the farms on which sampled flocks were reared. Each interviewee signed a written consent for participation. The Mississippi State University Institutional Review Board for the Protection of Human Subjects in Research provided approval for the survey instruments via administrative review on January 22, 2004 through IRB Docket #04-005.

The survey questionnaire underwent two pilot tests before the final edition was adopted. The first test was conducted with academic poultry veterinarians and the second with managers of a broiler complex in the area of the study [10]. In the survey, the interviewee was asked to specify for the sampled flock the lighting intensity maintained as the number of hours with full, dim, and black-out lights for each day of grow-out. A limitation of this categorization is that although the full and black-out lights are straightforward (*i.e.* the lights of maximum and minimum intensity, correspondingly), the dim lights may be interpreted as lights of any intensity between the two extremes.

The following parameters of the grow-out lighting were tested for associations with the burden of *Salmonella *in the flock: i) number of consecutive days at the start of grow-out when full lights were maintained for 24 hours per day, ii) day of grow-out when dim/black-out lights for ≥18 hours per day were introduced for the first time, iii) number of days with dim/black-out lights for ≥18 hours per day prior to the sample collection 1 week before the end of rearing, and iv) percentages of total hours of full, dim and black-out lights of total time of grow-out (days of grow-out multiplied by 24 hours). For the measurements of *Salmonella *in the flock 1 week before the end of rearing, the time percentages were approximate as they included the week between this sampling occasion and bird harvest.

### Statistical procedures

Each of the parameters of the grow-out lighting was tested for associations with each measure of *Salmonella *in the flock. Logistic regression was used, with the dependent variable (outcome) being the presence or absence of *Salmonella *in the samples from the flock, modelled with the events/trials syntax (*i.e.* number of *Salmonella*-positive samples/number of samples). To account for variability in the outcomes due to variation in the burden of *Salmonella *among grow-out farms within a complex, complexes within a company and between companies, the model incorporated hierarchically-structured random effects of the farms, complexes and companies. Each parameter of the grow-out lighting was tested in this multi-level mixed model as a single fixed-effects factor, and was considered to be associated with the outcome if *P *≤ 0.100. The 90% confidence level was used because of the observational nature of the study. If the fixed-effects risk factor was associated with the outcome, the significance of each of the three random-effects factors in the model was evaluated with a Wald-type test. The test statistic was calculated as [(parameter estimate/parameter standard error)^2^] and assumed to follow a Chi-square distribution with 1 *df *under the null hypothesis. A random-effects factor was considered to make significant contribution to variability in the outcome if *P *≤ 0.100. The models were fitted as generalized linear mixed models using the GLIMMIX procedure in SAS^® ^9.1 software for Windows (SAS Institute Inc., Cary, NC, USA).

## Results

No associations were observed between parameters of the grow-out lighting and probabilities of detecting *Salmonella *in ceca or crop of broilers 1 week before the end of rearing or upon arrival for processing, or on the broiler carcasses prior to immersion chilling. However, the associations were observed for detection of *Salmonella *on the exterior of birds 1 week before the end of rearing and upon arrival for processing, in the post-harvest drag swabs of litter, and on the broiler carcasses at the post-chilling point - the end of processing (Table 1). The parameters of the grow-out lighting associated with the outcomes of *Salmonella *in the flock were the percentages of total hours of full, dim and black-out lights of total time of grow-out, and the day of grow-out when dim/black-out lights for ≥ 18 hours per day were introduced for the first time. The variation in *Salmonella *burden among grow-out farms within a complex significantly contributed to variability in the outcomes in these models (in all cases *P *≤ 0.100). No such contribution was observed for the variation among complexes within a company or between companies (in all cases *P *> 0.500).

**Table 1:** Odds-ratios of detecting *Salmonella *in broiler flock and house litter depending on parameters of grow-out lighting.

		Parameter/its association with the outcome			
		
Outcome sample type	*n*	Parameter	Mean (range)	OR (Wald-type 95% CI)	*P*
Feathered carcass rinsates 1 week before the end of rearing	58	10% increase of the hours of full lights during grow-out	25.5% (6.5%-81.2%)	1.38 (0.98, 1.95)	0.061
	
	58	10% increase of the hours of black-out during grow-out	12.4% (0%-23.9%)	0.32 (0.09, 1.05)	0.060

Post-harvest drag swabs of litter from grow-out house	50	Day of grow-out when dim lights for ≥18 hours per day started	15 (3-29)	0.89 (0.78, 1.01)	0.065

Feathered carcass rinsates at arrival for processing	50	Day of grow-out when dim lights for ≥18 hours per day started	15 (3-29)	0.93 (0.85, 1.01)	0.098

Post-chilling carcass rinsates	54	10% increase of the hours of full lights during grow-out	25.5% (6.5%-81.2%)	1.31 (0.99, 1.74)	0.062
	
	54	10% increase of the hours of dim lights during grow-out	62.1% (3.2%-77.8%)	0.77 (0.56, 1.05)	0.091

Association between a parameter of grow-out lighting and an outcome was tested in a multi-level mixed logistic regression model that accounted for variation in the *Salmonella *burden among grow-out farms within a broiler complex, complexes within a company, and between companies. The lighting parameter was tested in this model as a single fixed-effects factor, and was considered to be associated with the outcome if *P *≤ 0.100; only such parameters are presented. In all these models, the variation among grow-out farms within a complex significantly (*P *≤ 0.100) contributed to variability in the outcome, but not the variation among complexes within a company or between companies (all *P *> 0.500). *n *- number of flocks.

## Discussion

In this analysis, a lower probability of *Salmonella *on both the exterior of broilers 1 week before the end of rearing and on the broiler carcasses at the post-chilling point of processing was associated with a longer relative duration (*i.e.* higher percentage of total time of grow-out) of reduced lights during rearing. The underlying mechanisms are likely to be interactive and may include: reduced stress and pecking behaviour, increased resting time, growth rates being better distributed throughout rearing period, improved walking ability, and improved resistance to infection in broilers with the longer reduced lights. Below we discuss how these effects may be impacting the burden of *Salmonella *in broiler flocks.

A lighting protocol combining full and reduced lights was shown to lead to the establishment of activity rhythms, increased sleep, reduced stress and better immuno-responsiveness in broilers compared to a protocol with 23 hours per day of full lights [11]. Reduced stress leads to a lower incidence of pecking behaviour. Decreased pecking and scratching were associated with reduced *Salmonella *presence in broilers in a prior field study [12]. Lower visual contrast during reduced lights may further decrease the stimulus to peck. Broilers have a higher number of uninterrupted resting periods with reduced lights, resulting in greater behavior synchrony in the flock [2]. The lighting protocol also affects the uniformity of broiler size within the flock throughout rearing [13]. A higher degree of uniformity in the case of more balanced periods of intense lighting may decrease the incidence of pecking due to the social ranking stimulus.

In an experimental study, reduced rates of mortality due to an infection (manifesting as peritonitis, polyserositis, hepatitis, septicemia, pericarditis, endocarditis or meningitis) were observed in broilers housed with 12:12 hours versus 20:4 hours per day of full and reduced lights, respectively [14]. All the birds were housed with 23 hours per day of full lights for the first 4 days of rearing, before the differential lighting programs were initiated. Reduced incidence of those infections may be suggestive of a higher immuno-competence of birds housed with the balanced lighting; there may be similar effects for *Salmonella*, although salmonellas of food-borne concern usually do not lead to a clinical disease in broilers. In another experiment, a higher peripheral blood T-cell proliferation response was detected in 6-week old broilers housed with intermittent lights compared to those housed with 23 hours per day of full lights (both groups were reared with 24 hours of full lights per day for the first 3 days; the birds were reared without a heat stress) [15]. However in another study no difference was detected in the heterophil:lymphocyte ratios in blood of 40 days old broilers reared with 18:6 versus 23:1 hours per day of the full and reduced lights, respectively [16].

Significant, but hard to interpret, interactions were reported between the effects of lighting, form of feed, and nutrient density in determining the quality of broiler leg bone (tibiotarsus) [14]. For the net effects on broiler walking ability, there was an interaction between the duration of full lights per day and bird gender. However the bone quality and the ability to walk were better in birds of either gender housed with the 12:12 compared to the 20:4 lights. For male broiler chickens, the effect of lighting on the strength of leg bones may additionally depend on the breed-strain of the birds [17]. Nonetheless, if the birds reared with more balanced lighting are better able to walk and spend less time sitting on the litter during the periods of full lights, this might lessen opportunity for horizontal transmission of *Salmonella *between the birds' exterior and the litter.

On the other hand, longer resting periods during the reduced lights may extend the opportunity for birds to 'sample' the litter through 'cloacal drinking'. (This refers to the uptake of particles from the environment by birds through a sucking movement of the cloacal lips, resulting in antigenic challenge of the bursa of Fabricius.) This may lead to the birds having higher immunity to *Salmonella*, and lower *Salmonella *intestinal carriage and shedding in faeces. This hypothesis fits with the observed associations between longer relative duration of reduced lights and lower probabilities to detect *Salmonella *on the exterior of broilers and on the carcasses during processing. However, the former was not associated with a decrease in *Salmonella *detection in ceca of broilers.

The prevalence of birds with (any) *Salmonella *in the ceca, crop or on the exterior, *Salmonella *presence in the litter, and the prevalence of *Salmonella*-contaminated carcasses during processing were evaluated in the flocks sampled in this study. The enumeration of *Salmonella *in individual samples was not done. The lighting practices may affect the quantities of *Salmonella *in ceca or crops and thus the prevalence of *Salmonella *on the carcasses, even though the prevalence of birds bearing *Salmonella *in ceca or crop during rearing was not apparently influenced. The associations between longer relative duration of reduced lights and lower probabilities to detect *Salmonella *on bird exterior and broiler carcasses could be indicative of those lighting-influenced reductions in the numbers of salmonellas in the organs of birds during rearing. Detailed quantitative data on how, and which, effects of the grow-out lighting influence the immune responses in broilers and how these affect the numbers of salmonellas in organs of birds are needed to test this hypothesis.

A recent study analysing the effects of lighting practices on broiler performance (in terms of the production indexes) showed that the effects differ depending on the market age of the birds [18]. For female broilers reared until 56 days old the negative effects of reduced early growth on feed conversion with longer reduced lights were compensated by a higher yield of legs and wings at the expense of breast yield by the market age [19]. No difference in cumulative feed conversion, but a reduction in breast meat yield was observed in broilers reared with longer reduced lights per day until 49 days old [16]. However, in broilers raised until 35 days or 40 days with relatively longer periods of reduced lights per day, the feed intake, feed conversion and body weight were reduced [13,17,20]. Broilers sampled in the present study were on average 56 days old at the time of harvest, but the market age ranged from 48 days to 61 days. Therefore it is impossible to interpolate what effects the grow-out lighting had on bird performance; the bird performance in turn could have impacted the burden of *Salmonella *in sampled flocks.

Lower risks of *Salmonella *on the exterior of broilers arriving for processing and in the post-harvest drag swabs of litter from the grow-out house were observed in the flocks in which reduced lights for ≥18 hours per day were introduced at a later day of grow-out compared to the other flocks. This lighting schedule was first started anywhere between the 3rd and the 29th day of rearing. Because of this large range and the variable total length of grow-out of sampled flock, the start-day variable was not 'standardized' for analysis. As discussed above, previous studies suggested that reduced lights in the beginning of grow-out lead to slower growth rates but better walking ability and reduced levels of stress in broilers, with feed conversion efficiency compensated at later stages of rearing (depending on the broiler market age). However, at least 6 hours of full lights per day during the first 21 days of grow-out are needed to avoid depression in growth of the birds [18]. Broilers exposed to less than 15 hours of full lights per day at this time can adapt to the darkness and became nocturnal [18]. Perhaps the latter was avoided by introducing reduced lights for ≥18 hours per day at a later day of grow-out, and this was associated with reduced risks of *Salmonella *on the exterior of broilers and the house litter. The condition and behaviour of birds in the beginning of rearing are especially important because an exposure to *Salmonella *at this time impacts their *Salmonella *status later on. Day-old (day-of-hatch) broilers exposed to as few as 100 salmonellas can be colonized and spread the pathogen to a group of birds by 3 weeks of age [21,22]. However, the effects of early exposure to *Salmonella *may be confounded by other factors and be undetectable by the time of processing (see for example Corrier *et al. *[23]). The results of the present analysis suggest that grow-out lighting is one of the confounding factors. The other confounders are likely to be further differences between grow-out farms, rather than more generalized differences between broiler complexes within companies or between companies (as *per *significance of the random-effects structure of the risk factor models).

Not only the relative duration of the full and reduced lights, but also how frequently the lighting intensity is changed during the day matters in terms of the effects on broiler growth, walking ability and mortality [24]. In the present study the lighting practices were not evaluated in sufficient detail to allow the relationships between the lighting intensity intervals and the burden of *Salmonella *to be tested.

Whether the grow-out lighting affects the ecology of other food-borne pathogens, *e.g. Campylobacter*, in broilers and whether it affects the distribution of such pathogens in other poultry are questions to be investigated.

## Conclusions

The results of this field study show that the lighting during broiler grow-out can impact the burden of *Salmonella *in the flock. Longer relative duration of reduced lights was associated with decreased detection of *Salmonella *on the exterior of birds 1 week before the end of rearing, and on the broiler carcasses at the post-chilling point of processing. Starting reduced lights for ≥18 hours per day later in the grow-out period was associated with decreased detection of *Salmonella *on the exterior of broilers arriving for processing, and in the post-harvest drag swabs of litter from the grow-out house.

## Competing interests

The authors declare that they have no competing interests.

## Authors' contributions

Design of sample collection: RWW, RHB, JAB, SAH and DM. Sample collection and processing: RHB, RWW and VVV. Survey design and implementation: VVV, RWW, SAH and DM. Survey data entry: VVV. Analysis: VVV and RWW. VVV and JAB drafted the paper; the other authors helped writing the paper. All authors read and approved the final manuscript.
